# Incidence and characteristics of candidemia in hospitalised patients with advanced HIV in the Médecins Sans Frontières (MSF) hospital in Kinshasa, Democratic Republic of Congo (DRC)

**DOI:** 10.1186/s12981-026-00890-5

**Published:** 2026-05-06

**Authors:** Chadrack Walo, Patrick Kisaka, Jean-Claude Mikobi Maleshila, Ghislain Mwenemboka, Fidèle Kutomisa, Ridi Fuka, Nadine Ntabugi, Judith Mulanga, Augustin Nzembomba, Astan Dicko, Alain Tshimungu, Charles Kasenda, Gisèle Mucinya, Fabiola Gordillo Gomez, Rosie Burton, Pilar Garcia-Vello, Agnese Comelli

**Affiliations:** 1 Médecins Sans Frontières, Kinshasa, Democratic Republic of Congo; 2 Médecins Sans Frontières, Middle East Medical Unit, Beirut, Lebanon; 3 Médecins Sans Frontières, MSF Academy, Brussels, Belgium; 4 Médecins Sans Frontières, Operational Research and Epidemiology Support Unit (LuxOR), Luxembourg, Luxembourg; 5 Brussels, Belgium

**Keywords:** Candidemia, Advanced HIV disease, LMIC

## Abstract

Candidemia is an underrecognised complication among patients with advanced HIV disease (AHD), particularly in low‑resource settings. We conducted a retrospective study at an MSF-supported hospital in Kinshasa (2022–2025) to describe the incidence, characteristics, and outcomes of candidemia. Among 3974 AHD admissions, 46 candidemia cases (1.2%) were identified, more than 80% of which were healthcare‑associated. Mortality was high (76.1%) and many deaths occurred before microbiological confirmation. Classical risk factors, such as indwelling catheters and parenteral nutrition, were uncommon, suggesting alternative pathogenic mechanisms. Findings highlight the need for improved diagnostics and access to antifungal susceptibility testing.

**Supplementary Information:**

The online version contains supplementary material available at 10.1186/s12981-026-00890-5.

## Introduction

*Candida albicans* is classified by the World Health Organization (WHO) as a critical fungal priority pathogen [1]. Globally, invasive fungal infections are rising, and candidemia carries high mortality, estimated at 20–50% even with appropriate treatment [1]. In low‑income countries (LIC), diagnosis and management are further hindered by limited diagnostics, restricted antifungal availability, and emerging resistance.

Fungal diseases receive limited attention and funding, resulting in major gaps in epidemiological data and challenges in estimating the global burden of candidemia, especially in LIC. Evidence is particularly scarce for people living with HIV. Few studies have addressed invasive *Candida* spp. infections in patients with advanced HIV disease (AHD) in such settings [2, 3]. AHD is characterised by a CD4 count below 200 cells/mm3 or a WHO HIV clinical stage of 3 or 4. Although WHO clinical stage 3 or 4 includes oral candidiasis and oesophageal candidiasis, HIV infection is not traditionally considered a major risk factor for invasive *Candida* infections [4]. However, AHD presents multiple predisposing factors for candidemia, including low CD4 count, malnutrition, cachexia, *Candida* colonisation, neutropenia or neutrophil dysfunction, exposure to broad-spectrum antibiotics, prolonged hospitalisation, central venous catheter (CVC) use, and increased intestinal translocation [5, 6].

Within the Médecins Sans Frontières (MSF) HIV/AIDS project at Centre Hospitalier de Kinshasa (CHK), extensive experience in the care of people with AHD, combined with microbiological capacity since 2022, provides a unique opportunity to address this evidence gap.

This study aims to describe the incidence, clinical and management characteristics, and outcomes of candidemia among hospitalised patients with AHD at an MSF-supported hospital in Kinshasa, Democratic Republic of Congo.

## Materials and methods

A retrospective observational study was conducted between September 2022 and April 2025 at the MSF-supported Centre Hospitalier de Kinshasa (CHK). Adult patients with advanced HIV disease (AHD; CD4 < 200 cells/mm^3^ or WHO stage 3–4) and candidemia, defined as at least one blood culture positive for *Candida* spp., were included.

Blood culture collection criteria utilised in this facility are presented in table S1 (see supplementary).

When yeast was detected on Gram stain, clinicians were informed, and an India ink test was performed to differentiate *Candida* spp. from *Cryptococcus* spp. If negative, candidemia was confirmed. Species identification and antifungal susceptibility testing (AST) were not available. Cryptococcus antigen test is available.

CHK provides comprehensive AHD care, including intensive care unit (ICU) admission, oxygen support, intravenous therapies via peripheral access and increased nurse: patients ratio in the ICU, but lacks central venous catheters, parenteral nutrition, mechanical ventilation, and advanced imaging.

Discharge diagnoses are determined by the clinicians who compile the discharge report and there is no specific rule to rank them.

Routinely collected hospital data were extracted from WHONet and medical records. Incidence rates were calculated using patient-days from all adult AHD admissions. Data were merged using patient identifiers and analysed with STATA v17. Continuous variables were summarised using median and interquartile ranges (IQR), and categorical variables using frequencies and proportions. Survival was estimated using the Kaplan–Meier method; correlation analyses were not performed due to the small sample size.

## Results

Forty-six patients with candidemia were registered among 3974 patients (1.2%) admitted during the study period.

**Fig. 1:**
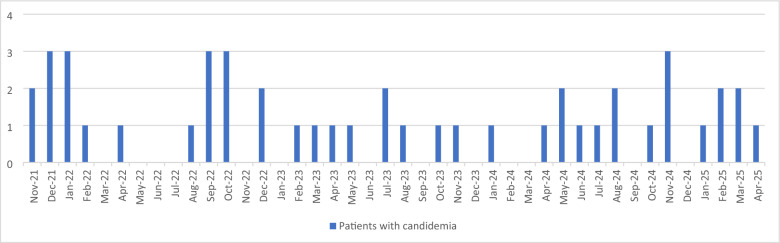
Candidemia cases over time (September 2022 to April 2025)

The distribution of case counts is shown in Fig. 1, and no cluster of cases seems to be present.

The main characteristics of the population are reported in Table 1.

**Table 1:** Candidemia patients’ characteristics

Characteristics	Candida cases (n = 46)
Female, n (%)	31 (67.4%)
Age, median (IQR)	41.6 (35–51)
CD4 < 200, n (%)	33 (82.5%)^a^
VL > 1000 copies/uL, n (%)	30 (73.2%)^b^
BMI < 18.5, n (%)	23 (63.9%)^c^
ARV compliance in the last 6 months^d^, n (%)	21 (45.7%)
WHO stage, n (%)	
- 3	5 (10.9%)
- 4	41 (89.1%)
Antibiotic therapy in the 30 days prior to admission, n (%)	32 (69.6%)
History of oral/oropharyngeal candidiasis, n (%)	4 (9.5%)
GI signs and symptoms at admission, n (%)	15 (32.6%)
Discharge from hospital in previous 30 days, n (%)	25 (55.7%)
ICU admission during the same hospitalisation, n (%)	28 (63.6%)
Hospital stay (days), median (IQR)	9 (5–19)
Antibiotic treatment at *Candida* diagnosis, n (%)	40 (87%)
Steroids before *Candida* diagnosis, n (%)	15 (32.6%)
Duration of steroid therapy before candidemia diagnosis, median (IQR)	3 (1–8)

ATB: antibiotic, GI: gastro-intestinal, ICU: intensive care unit, BMI: body mass index, IQR: interquartile range, VL: viral load

^a^information available for 40 patients

^b^information available for 41 patients

^c^information available for 36 patients

^d^self reported by the patient

Frequent co‑morbidities included central nervous system (CNS) tuberculosis (60.9%), neurotoxoplasmosis (45.7%), renal disease (47.8%) and pneumocystosis (43.5%).

Regarding the timing of diagnosis relative to the date of admission, in 8 cases (17.4%), the candidemia was diagnosed within the first 48 h. For the remaining 38 (82.6%), candidemia was healthcare-acquired: 24 patients (63.2%) developed the infection after 48 h from the hospital admission (hospital-acquired infections) and 14 (36.8%) reported a recent hospitalisation (discharge within 30 days).

The incidence rate of hospital-acquired candidemia was 3.9/10,000 patient-days (95% CI 2.5–5.7).

Among the 24 hospital-acquired infections, the median time from admission to positive blood culture (BC) collection date was 8.5 days (IQR 5.5–13).

From the laboratory side, it took a median of 2 days (IQR 2–3) from BC collection date to the stain being reported positive for yeasts. Confirmation of *Candida* sp isolate took a median of 3 days (IQR 3–4).

### Mortality and patient management

**Fig. 2:**
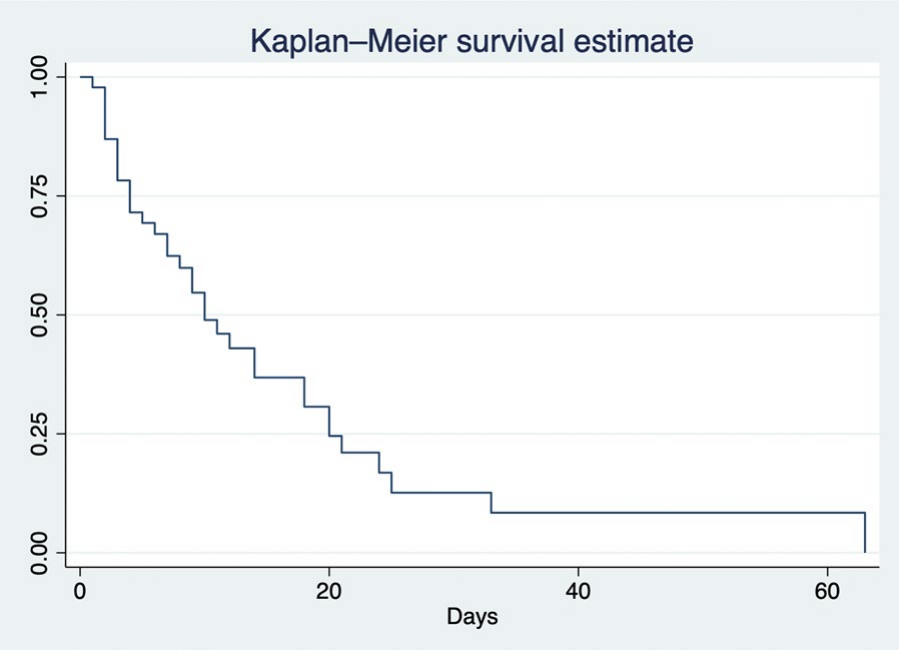
Kaplan–Meier survival curve

Overall, in-hospital mortality was 76.1% (35/46). The Kaplan–Meier curve is shown in Fig. 2. Median survival from admission was 8 days (IQR 3–18). Twenty-two patients out of 46 (47.8%) died within 2 days of the positive BC collection date.

Previous admission in ICU occurred in 63.6% of candidemia cases, and 83.3% of those who died had a previous admission in ICU.

Steroid use before candidemia occurred in 32.6% of cases, mostly among those who died (14/15). Median duration of steroid treatment before candidemia was 8 days (IQR 3–11) among those who died early after presenting with candidemia (i.e. within 2 days from the positive BC collection date). By contrast, patients who recovered from candidemia or who survived longer before death, received a shorter duration of steroid treatment (median of 1 day, IQR 0–1). The most common recommendations for steroids were as part of the treatment for *Pneumocystis jirovecii* Pneumonia (PCP), severe immune reconstitution inflammatory syndrome (IRIS) and CNS tuberculosis.

Eighty-seven per cent of patients were receiving oral or IV antibiotic treatment at the diagnosis of candidemia. Most commonly with trimethoprim/sulfamethoxazole (58.7%), used in all cases either as prophylaxis or treatment for pneumocystosis or toxoplasmosis, meropenem (50%), and vancomycin (17.4%). All patients who died were receiving antibiotics at diagnosis, compared to none of the survivors.

Twenty-three patients (51.1%) received antifungals: 19 (82.6%) liposomal amphotericin B, and 4 cases (17.4%) with fluconazole directly. In two cases, liposomal amphotericin B was initiated on the date of blood culture collection due to concomitant suspicion for cryptococcal meningitis. Fourteen (60.9%) began antifungals the same day as the positive stain, 4 cases (17.4%) the next calendar day and the treatment was started after 2, 3, and 4 calendar days for the remaining 3 patients, respectively. Of the 22 (48.9%) untreated patients died before the BC results were available. In one case, we weren’t able to retrieve the information about antifungal treatment.

Mortality among those who received antifungal treatment was 65.2% (15/23).

### Candidemia documentation

Candidemia was documented in discharge reports (5 diagnoses per patient accepted) in 30.4% (14/46) of patients with known candidemia and in 28.6% (10/35) of patients who died. Three patients had candidemia as first diagnosis.

## Discussion

In this study, we describe the clinical characteristics and outcomes of patients with advanced HIV disease diagnosed with candidemia. Although only 1.2% of the patients hospitalised in the study period developed candidemia, the true proportion is likely to be underestimated due to the limited sensitivity of blood cultures, particularly in severely ill and profoundly immunosuppressed patients.

Mortality in this cohort was extremely high (76.1%), including among patients who were treated promptly with antifungals (65.2%), exceeding rates reported in high- and middle-income settings: cohorts from Italy, South Africa, and France reported mortality ranging from 38 to 59% [2, 7, 8].

Given the profound immunosuppression in this population, with the 80% of patients having CD4 < 200 cells/µL, mortality cannot be attributed solely and primarily to candidemia. Even among those who received antifungal treatment, mortality exceeded 60%, suggesting that candidemia may be a marker of critical illness rather than an isolated cause of death. This is supported by the high prevalence of prior ICU admission and ongoing antibiotic therapy among deceased patients, reflecting the complexity of attributing mortality AHD. However, it is also important to highlight the lack of availability of echinocandins, which are recommended in most guidelines as first-line treatment for candidemia. Indeed, amphotericin B and fluconazole are more widely available due to their use in cryptococcal meningitis and non-invasive candidiasis. Growing data on candidemia in individuals with AHD and other immunosuppressed populations could be significant for advocating broader and more affordable access to echinocandins, which, notably, are included in the WHO Essential Medicine list [9].

Candidemia was substantially underreported in clinical documentation: it appeared in discharge diagnoses in 40.4% of cases. Under‑recognition likely contributes to underestimating the burden of invasive fungal infections, highlighting the need to better integrate laboratory-confirmed results into clinical documentation in LMICs. A focused investigation into the causes of this underreporting would be important to guide corrective measures.

Classical risk factors for candidemia, such as intravascular devices and parenteral nutrition [10] appear not to be relevant in this study population because they are not available in the hospital. Steroid exposure prior to candidemia diagnosis was limited both in frequency and duration. Notably, 17.4% of cases presented with candidemia within the first 48 h of admission, consistent with reports from other AHD cohorts [2, 7]. This suggests alternative pathogenic mechanisms- particularly intestinal fungal translocation in severely immunosuppressed patients [6], rather than truly community acquisition. For this reason, we avoided classifying early-onset cases as community-acquired.

A significant proportion of patients died before preliminary microbiological results were available, preventing initiation of antifungal therapy. Although laboratory turnaround was relatively rapid, earlier diagnosis—potentially through molecular or non-culture-based methods—might be required to improve outcomes in this highly vulnerable population. Unfortunately, the likelihood of these tests being available in LIC is very low at present, given the lack of access even to basic microbiology culture.

The absence of antifungal susceptibility testing limited assessment of resistance, an emerging issue in people with AHD frequently exposed to antifungals [11]. Existing African data remain scarce and derive largely from non‑invasive infections, with resistance rates to azoles ranging from 0 to 70% [12]. This lack of data on invasive candidiasis underscores the need for expanded access to species identification and antifungal susceptibility testing in LMICs, to better inform treatment guidelines and antifungal stewardship strategies.

This study has limitations, including its retrospective, single‑centre design, small sample size, lack of a comparator group, and difficulty in attributing the cause of death. Furthermore, access to species level identification and antifungal susceptibility testing would have enhanced understanding of the problem. Nonetheless, it provides rare data from a setting with limited diagnostics. Strengthening surveillance of invasive fungal infections, as urged by WHO, remains essential. Sharing evidence from centres where such diagnostics are available is crucial to improving understanding of invasive fungal infections in LICs. In this regard, our findings align with the recent WHO call to strengthen data on priority fungal infections, including their burden, complications, and mortality [1].

In conclusion, we reported high mortality in patients with AHD and candidemia, despite antifungal treatment. This highlights candidemia as a rare but extremely severe and life-threatening, underrecognised complication in people with AHD in LMICs and the need for better diagnostic access, including *Candida* identification and antifungal testing.

## Supplementary Information

Additional file1 (DOCX 18 KB)

## Data Availability

The dataset is available with the corresponding author and can be shared upon reasonable request.
