# Supplementary material for: Incidence and characteristics of candidemia in hospitalised patients with advanced HIV in the Médecins Sans Frontières (MSF) hospital in Kinshasa, Democratic Republic of Congo (DRC)
Source: AIDS Res Ther. 2026 May 6;23:69. doi: 10.1186/s12981-026-00890-5 (PMC13359897; doi:10.1186/s12981-026-00890-5)
Supplement: Supplementary file 1 — Additional file1 (DOCX 18 KB) [file 12981_2026_890_MOESM1_ESM.docx]

Supplementary

Table S1. Criteria for blood culture collection used in the MSF-supported Centre Hospitalier de Kinshasa (CHK)

| **Patients presenting to the Emergency Department** |
| --- |
| Signs of sepsis with/without a clear source (e.g., pneumonia, meningitis) |
| Fever (Axillary T◦ ≥ 38◦C) **OR** Hypothermia (Axillary T◦ ≤ 36◦C) **AND** at least one of the following signs:  - Hypotension (systolic blood pressure =< 100 mmHg) or clinical signs of shock, such as capillary refill time> 3 sec, signs of poor peripheral tissue perfusion  - Confusion or altered state of consciousness (Glasgow score < 15)  - Increased respiratory rate (>= 22 per minute)  **OR** another serious infection, e.g. severe malaria, typhoid is suspected  **OR** medical decision with clear documentation of the indication in the patient's record |
|  |
| **Patients hospitalized for more than 48 hours** |
| Therapeutic failure with signs of sepsis (see above) or septic shock despite well-conducted antibiotic treatment  **OR**  Healthcare-associated infection: appearance after 48 hours of hospitalisation of signs of sepsis (see above), whether or not the patient is on antibiotics or has already been diagnosed with another disease. |
|  |
| **Patients with unexplained fever with no obvious focus and lack of response to empiric treatment for opportunistic infections** |
